# The Ecologist's Career Compass: A game to explore career paths

**DOI:** 10.1002/ece3.9259

**Published:** 2022-09-15

**Authors:** Yuval Itescu, Maud Bernard‐Verdier, Simon S. Moesch, Agata Mrugała, Kinga Mrugała, Camille L. Musseau, Jonathan M. Jeschke

**Affiliations:** ^1^ Leibniz Institute of Freshwater Ecology and Inland Fisheries (IGB) Berlin Germany; ^2^ Institute of Biology Freie Universität Berlin Berlin Germany; ^3^ Geography Department Humboldt Universität zu Berlin Berlin Germany; ^4^ Freelance Artist Łódź Poland

**Keywords:** applied games, card games, career development, ecology job market, individual career paths, mentorship

## Abstract

One of the most challenging endeavors for students is choosing a career path that best fits their interests, wills and skills, and setting their professional goals accordingly. Such decisions are often made from within the culture of academia, in which mentors and peers are mainly familiar with the academic job market and lack the knowledge necessary to consult about other types of careers. We aimed to address this gap for ecology and related fields by creating an engaging and effective tool to help students and professionals to familiarize themselves with the diversity of potential career paths available to ecologists. The tool is an applied card game – the *Ecologist's Career Compass* – which is provided here freely*.* The game is played as a trump card game and includes 33 cards, each representing a combination of one of four job‐market sectors and one of nine types of positions. Each card indicates the level of seven skill categories required to likely be hired and succeed in the focal position at the focal sector, as well as more specific examples for typical jobs in the focal combination. The information in the game largely relies on input from a global survey we conducted among 315 ecologists from 35 countries. While the challenges faced by early‐career ecologists in developing their professional path are substantial and diverse, this game can assist in gaining a broad comparative overview of the whole ecology job market and the skills required to likely excel in different paths. We hope this applied game will act as a conversation starter about the diversity of aspirations and opportunities in ecology classrooms and labs.

## Graphical abstract

We conceived and designed an applied card game that provides players with a fun tool for acquiring knowledge about the possible career paths in ecology across all employment sectors and the skills required to likely be hired and succeed in each path. We aimed to build a tool that will also aid academic mentors in advising their ecology students about career development.

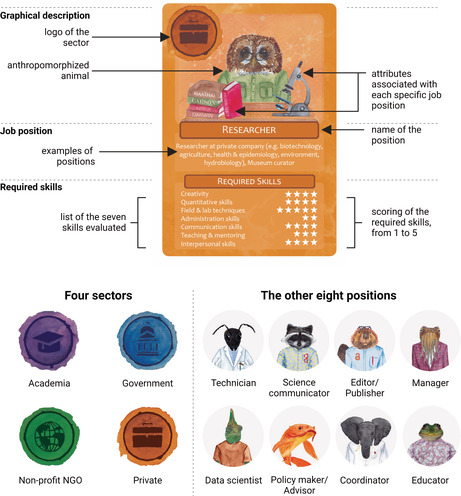

## 1 INTRODUCTION

Choosing a career that will best fit one's personal aspirations, talents and values, in addition to being enjoyable and fulfilling, is a challenge (Sen, 2021). Students in many fields in the higher education system are often encouraged to pursue an academic research career (Woolston, 2015) and face the unjustified perception that leaving academia is a failure (Kruger, 2018), although the job market for graduates is far from being limited to academic research positions. Understanding which career paths exist in one's training field, and which skills are required to likely be hired and successful in a chosen path, are keys in this search process; they are also necessary prerequisites to being able to fully incorporate the many considerations involved in career‐path choices (Bourne, 2017; Searls, 2009). Yet such information is difficult to find, and there are large gaps in knowledge and awareness of the diversity of potential career paths among young academics, especially at the early stages of their higher education.

Academic teachers and research group leaders are one of the primary and most influential sources from which students seek advice in setting their goals and designing their careers (Nora & Crisp, 2007). However, because these academic mentors are usually acquainted mainly with the academic job market, they often cannot provide proper guidance, direction and relevant knowledge about non‐academic careers for their students. This impedes their mentorship in this regard and prevents students from enjoying an adequately informed perspective when searching for the career path that would best fit their skills and wills. Academic peers, which are another important source for information and consultation, in many cases are similarly bounded to their own experiences, which also stem from the academic culture that considers mainly the academic job market. Furthermore, independent access to professional literature is seldom practiced by early‐stage students, either because of awareness gaps, lack of proper training or practical difficulties, making the role of experienced academics in directing and introducing students to the available relevant information vital.

In the field of ecology and evolution, most PhD graduates are not employed in faculty positions (Hampton & Labou, 2017). Early‐career ecologists, such as most of us authors (and early‐career academics in general, especially those coming from historically marginalized communities), face immense personal and professional struggles in developing their academic career. The increasing mismatch between the number of ecology graduates and the number of available faculty positions, and the growing recognition of the need to assist graduates in finding their path in the job market have motivated the publication of several sources of information about non‐academic ecology careers in recent years. These include essays describing individual examples of potential career paths (e.g., Klemow et al., 2017 and the papers in this series), skills required in non‐academic careers (e.g., Blickley et al., 2013), and the transferability of academic skills to non‐academic sectors (e.g., Roberts et al., 2021). However, there is, to our knowledge, currently no comparative tool representing the diversity of positions in ecology to help prospective ecologists choose their path.

Motivated by our own personal experience with such challenges, we addressed this gap by developing an applied trump‐like card game that allows players to explore, in a social and entertaining way, the diversity of potential career paths in ecology and related fields, and the skills required to likely be hired and excel in each path. The ability of games to enrich and enhance the learning experience and performance of players, and stimulate discussion around the topics of the game, is well‐substantiated (Subhash & Cudney, 2018). This seems particularly true for card games (Barnes, 2020; Franklin et al., 2003), including trump card games (Chiotaki & Karpouzis, 2020; Spandler, 2016) where players become familiar with the information on the cards.

## 2 THE ECOLOGIST'S CAREER COMPASS

**FIGURE 1:**
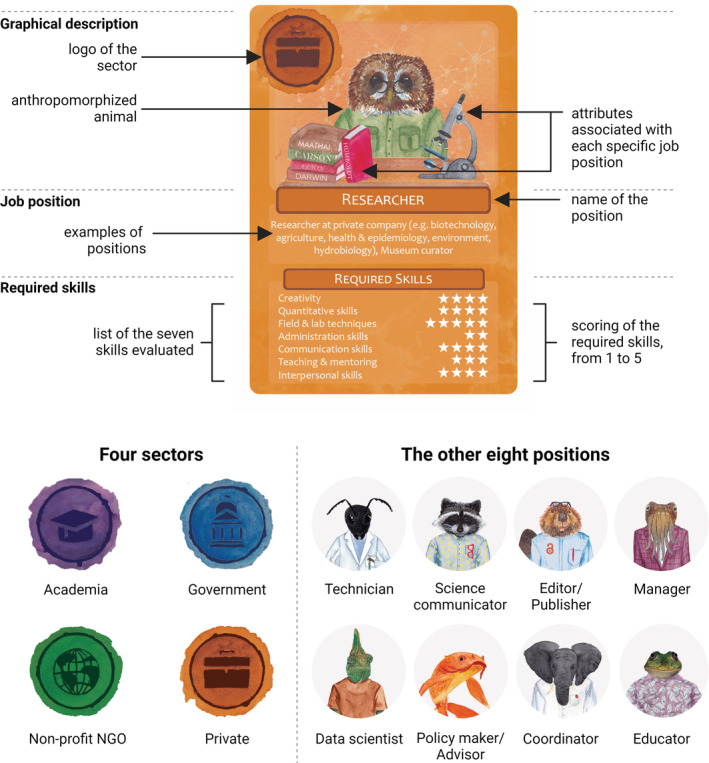
Design of the cards. The example card shown on the top is *Researcher* in the *Private sector*. All four sectors and the other eight types of positions are shown on the bottom. As ecologists, we chose animals from a broad range of taxa as a fun and artistic way to represent the diversity of positions, and assigned them randomly to avoid as much as possible implying certain characteristics for the positions. The required skills for each position in the different sectors are scored on the cards (see Appendix S1). The figure was created with BioRender.com.

The card game is called the *Ecologist's Career Compass*. We freely provide it in the Appendix S1, for self‐printing, with no commercial interest. It is mainly intended for BSc and MSc students, but it can also be helpful for high‐school students as well as for PhD candidates and postdocs. The *Ecologist's Career Compass* includes 33 cards (see example in Figure 1) and follows the rules of trump card games with a few adjustments to improve its flow and attractiveness (see game's rules in Appendix S1). Each card represents one of nine position types (see Box 1) in one of the four job market sectors: academia, government, non‐governmental organizations (NGOs), and private (three of the sector‐position combinations do not exist in the real world, as far as we know, which is why there are 33 cards instead of 36). Finally, each card shows the importance of seven skills for the focal sector‐position combination on a scale from 1 (low) to 5 (high).

BOX 1 **Descriptions and examples of the different types of positions in the game**

**Table:** 

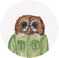	**Researcher**, e.g., university professor or scientist at a research institute, NGO or company – researchers lead and carry out research projects (either independently or commissioned by the employer) and are responsible for the execution of most or all research steps (e.g., study design, data collection, analyses, producing and publishing research reports). Other potential tasks of researchers include fundraising, communicating research to peers and the public, serving as expert and mentoring students and early‐career researchers
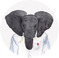	**Coordinator**, e.g., head of administration or project coordinator – coordinators are responsible for the administrative and overall functioning of processes, projects, and schemes commissioned by their employer
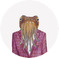	**Manager**, e.g., project manager or manager of a field station, nature reserve or laboratory – managers are responsible for the operative functioning of projects and schemes commissioned by their employer, including decision‐making of professional, economical, and human‐resource aspects
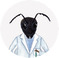	**Technician**, e.g., technician in the laboratory or field, research engineer, toxicologist or GIS technician – technicians provide operative services and technical expertise to their employer
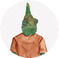	**Data scientist**, e.g., environmental informatician, GIS analyst or bioinformatician – data scientists provide advanced analytical and programming services, and data‐informed insights for promoting the objectives of their employer
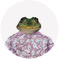	**Educator**, e.g., lecturer or teacher, nature educator or guide/educator in national park, museum, zoo or aquarium – educators teach and develop the learning experience of their audience, provide information on whole topics (rather than single focal studies), build learning modules and materials, and train their audience in the topics of interest of their employer. They target specific groups (e.g., students or visitors of nature‐oriented places)
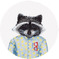	**Science communicator**, e.g., science journalist, nature filmmaker, technical writer, public relations expert – science communicators transfer scientific information in an easy‐to‐understand and attractive way, raise awareness for scientific issues, provide exposure to scientists and the scientific process, and build understanding and appreciation of scientific discoveries and advancements. They target the general public
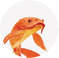	**Policy maker/advisor**, e.g., environmental consultant, science diplomat or lobbyist – they use their knowledge to examine the information available and provide recommendations for their employer, or assess and monitor aspects that relate to their employer's objectives
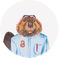	**Editor/publisher**, e.g., scientific editor or copy‐editor – they handle the procedures of scientific publications on behalf of their employer, and provide professional services for assessing and improving their quality

We believe that the information in the game represents the general ecology job market in many countries. We assessed the diversity of positions and evaluated the importance of a set of skills for each sector‐position combination in a multi‐step process. First, we collected information that appeared in job advertisements, career advice portals, online personal stories, and other publications about careers in ecology, to map which positions exist in the ecology job market in different countries. We also grouped different specific skills that appeared in the information we collected as essential job requirements or advantages, into seven general skill categories: creativity, quantitative skills, field and lab techniques, administration, communication, teaching and mentoring, and interpersonal skills. Second, we scored each of the seven skill categories in each sector‐position combination. In this step, we first assigned each sector to a different co‐author who reviewed the available information and determined the preliminary scores for the skills required for the different positions in the focal sector. Each score reflected the described level of importance and relative frequency of mentioning of a focal skill category or more specific abilities falling under a certain skill category as qualification requirements in the different sources of information about positions in a focal sector (e.g., job advertisements, blogs, literature, etc.). Then, we engaged in group discussions about each individual score to reach a consensus among us. In the third step, to substantiate our own preliminary scores, we conducted an anonymous global online survey via soscisurvey.com, which we distributed with an open call through popular social‐media platforms such as Twitter and Facebook, and professional networking mailing lists of ecologists in several countries. The survey was open from 26 March to 3 May 2021. The 315 participants represented 35 countries (in all continents) and 30 sector‐position combinations (division of participants by sectors: 182 academia, 53 government, 43 NGOs, and 37 private; division by position type: 182 researchers, 31 coordinators, 19 technicians, 6 data scientists, 22 educators, 15 policy makers/advisors, 2 editors/publishers, 31 managers, and 7 science communicators). All participants had at least a Bachelor's degree (2 BA, 5 BSc, 104 MSc, 204 PhD). We asked them to provide anonymous details about themselves and their current job: level of academic training, the country in which they work, and the sector, position type, and detailed title of their position. We also instructed them to score the importance of the seven skills for their current job via a dedicated continuous scale bar moving from 1 to 5. Finally, we compared the average scores from the survey with our previous assessments, discussed each case of discrepancy between our score and the survey‐based score (i.e., cases where the scores differed by two points or more out of five), and amended scores as needed. In this step, we also considered the number of people who scored each sector‐position combination, giving more weight compared to our own judgment if this number was high. The survey input confirmed our preliminary job‐market mapping: no new position type was suggested.

## 3 A TOOL FOR TEACHING AND MENTORING STUDENTS

The game provides academic group leaders and teachers in ecology and related fields an assisting tool in their efforts to advise their students and mentees about career paths. By playing it, aspiring or early‐career ecologists gain a fun way to familiarize themselves with the field's job market and benefit in the job search process by targeting positions that fit their skills and interests. Being a socializing tool by default, the game also acts as a conversation starter between students, their peers and mentors, and can stimulate the exchange of ideas, questions, and experiences about careers in ecology among players. The game is not limited to people currently in academia, and could furthermore support ecologists working outside academia who wish to explore new possibilities for their career. It can ideally be played as part of research group meetings, career development workshops and courses, social gatherings of students, and even as a fun activity during lunch breaks.

When academic group leaders and teachers use the *Ecologist's Career Compass* with their students, we suggest discussing challenges and opportunities of different career paths. Senior academics are well aware of them for academic positions, and we strongly encourage to share both of them with students and mentees. In our experience, the opportunities and benefits of academic life are indeed frequently shared with students. However, it is important to also present other career paths on an equal footing, and acknowledge and discuss openly the multiple reasons, systemic and personal, why an academic career path is not the best fit for everyone. First, the uncertainty and stress involved in obtaining permanent academic employment, fueled by the shortage of such positions in many countries, push trained and talented young scholars to consider alternatives (Gould, 2015). Second, academic life can be an emotional rollercoaster, where one has to deal with repeated rejections and the impostor syndrome (Jaremka et al., 2020), to the point that many young academics experience mental health crises (Evans et al., 2018; Woolston, 2018). Third, maintaining a healthy work‐life balance, which many consider a key priority in career choices, is notoriously challenging in often stressful academia (Bartlett et al., 2021). Fourth, other job sectors may appeal more than academia considering employment conditions, job security, and responsibilities. Finally, many young scholars are simply passionate about their training field without necessarily aspiring for an academic life of teaching, grant writing and research. Nevertheless, each career path offers challenges and opportunities. It can thus be very useful to invite professionals with the relevant job experience to share with students which challenges they encountered and which benefits come with the career path that they have chosen.

An applied game such as the Ecologist's Career Compass cannot (and is not aiming to) solve all the challenges that early‐career ecologists face. However, we found from our own experience designing it, and from feedback received by players participating in pre‐tests of the game that we performed, that becoming more familiar with the vast range of opportunities for ecologists helps to change perspectives and shine a light on new potential solutions for the problem of finding a career path that will best fit someone. We are therefore confident that the game can be an effective tool in this regard, and achieve its goal in several ways: (1) Opening players' minds about career paths that were not previously considered by them. (2) Providing a comparative basis for navigation in the ecology job market, while allowing to prioritize certain directions in the job search based on already acquired skills. (3) Acquainting players with likely skill requirements of certain career paths that one can opt to improve in order to pursue a desired path. (4) Motivating a more in‐depth investigation of potential career paths. (5) Serving as a reference for discussions about careers in ecology and the breadth of this job market.

In conclusion, we encourage senior ecologists to introduce the *Ecologist's Career Compass* to their students and mentees, help them to broaden their knowledge about different career options in ecology and related fields by playing this applied game, use it to inform their goal setting, and accordingly support them to build the skills that qualify them for their preferred career path(s).

## AUTHOR CONTRIBUTIONS

**Yuval Itescu**: Conceptualization (equal); Investigation (equal); Project administration (equal); Visualization (equal); Writing – original draft (lead); Writing – review & editing (equal). **Maud Bernard‐Verdier**: Conceptualization (equal); Investigation (equal); Project administration (equal); Visualization (equal); Writing – review & editing (equal). **Simon S. Moesch**: Conceptualization (equal); Investigation (equal); Project administration (equal); Visualization (equal); Writing – review & editing (equal). **Agata Mrugała**: Conceptualization (equal); Investigation (equal); Project administration (equal); Visualization (equal); Writing – review & editing (equal). **Kinga Mrugała**: Visualization (lead). **Camille L. Musseau**: Conceptualization (equal); Investigation (equal); Project administration (equal); Visualization (equal); Writing – review & editing (equal). **Jonathan M. Jeschke**: Conceptualization (equal); Investigation (equal); Project administration (equal); Visualization (equal); Writing – review & editing (equal).

## CONFLICT OF INTEREST

The authors have no financial or non‐financial interests that are directly or indirectly related to the work submitted for publication.

## Supporting information


Appendix S1

## Data Availability

All relevant data associated with this study is available in the main text and Appendix S1.
