# Supplementary material for: The Ecologist's Career Compass: A game to explore career paths
Source: Ecol Evol. 2022 Sep 15;12(9):e9259. doi: 10.1002/ece3.9259 (PMC9478519; doi:10.1002/ece3.9259)
Supplement: Supplementary file 1 — Appendix S1 [file ECE3-12-e9259-s001.pdf]

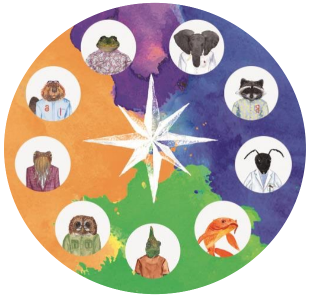

The Ecologist’s Career Compass

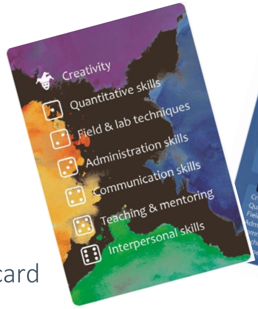

Joker card

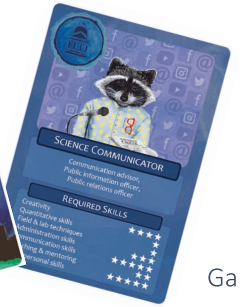

Game card

Number of players: 2 or more

Material: 33 career cards, 7 joker cards and a six-sided dice

Goal: Collect the highest number of cards. The game ends when one player has no card left. In a two-player game, the other player wins. In a multiplayer game, the player with the highest number of cards wins.

Game rules:

The game is similar to an ordinary **trump card game**, where at each round the players match skill-for-skill and the highest skill score wins. Unlike classic trump games, competing skills in this game are not selected by the players, but by a roll of the dice.

Start of the game: All 33 career cards are shuffled and dealt face down. The players take their cards as a closed package in their hand, so that no one else can see the top card. Before beginning the game, a **joker skill** is selected. This skill is used in case of a tie between players: the joker skill will determine who wins the round (see below for an example). This joker skill can be selected in the beginning of the game by picking one of the seven joker cards (either randomly or as wished by the players).

Example round: The players pick *Creativity* as the joker skill. The dice is thrown and shows a one, which in this case means the category *Quantitative skills*, thus the players should read out the skill level for this category on their top card. If two or more players have an equal skill level, then these players read the skill level for *Creativity* (the joker skill in this example). The player with the highest skill level receives the card(s) of the other player(s). Then the dice is thrown again ...

What happens if the skill levels for the joker skill are also equal? In such a case, the top cards of all players are thrown in the middle to form a stack. Those players who had the same highest skill level for the joker skill now continue to play for the stack with the next card in their hands. Once the stack is won by one of the players, the game continues as described above.

Players who have only three cards left can choose which of the cards to take, so under this special situation not only the top card is relevant but all three remaining cards. In this case, the player with only three cards left can choose not only which card to read but also which card to give away if losing the round, and the second can be different than the first.

This game is published under the CC BY-SA 4.0 licence:
Bernard-Verdier, M.; Itescu, Y.; Moesch, S.S.; Mrugała, A.; Mrugała, K.; Musseau, C.L.; Jeschke, J.M. 2022.
The Ecologist's Career Compass. Berlin, Germany.

The game emerged from discussions in our Ecological Novelty group in Berlin on ecological career paths. All of us contributed equally to it, while Kinga Mrugała artistically designed the cards. If you would like to contact her, e.g. for another art-design project, you can reach her under: kinga.mrugalaa@gmail.com

Additional information is provided in an accompanying paper:
Itescu, Y.; Bernard-Verdier, M.; Moesch, S.S.; Mrugała, A.; Mrugała, K.; Musseau, C.L.; Jeschke, J.M. 2022.
The Ecologist's Career Compass: a card game to explore career paths. *Ecology and Evolution*.

If you have further questions about the game, you may contact any one of us. Only for simplicity do we only give Jonathan Jeschke’s e-mail here: jonathan.jeschke@igb-berlin.de

FRONT SIDE

THE

ECOLOGIST'S CAREER COMPASS

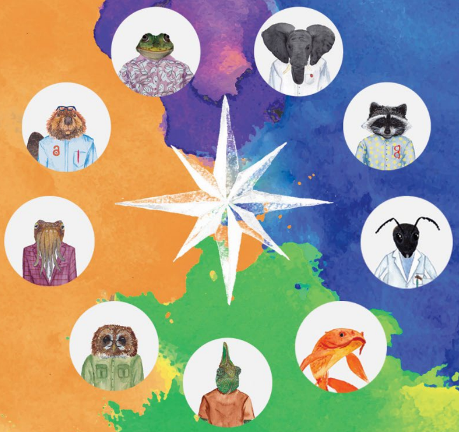

THE

ECOLOGIST'S CAREER COMPASS

MAUD BERNARD-VERDIER

YUVAL ITESCU

SIMON S. MOESCH

AGATA MRUGAŁA

KINGA MRUGAŁA

CAMILLE L. MUSSEAU

JONATHAN M. JESCHKE

IGB
Leibniz Institute of Freshwater Ecology and Inland Fisheries

Freie Universität Berlin

THE

ECOLOGIST'S CAREER COMPASS

SHORT RULES

1. GOAL: COLLECT THE MOST CARDS!
2. DISTRIBUTE ALL CAREER CARDS FACE-DOWN
3. PICK ONE OF THE SEVEN JOKER CARDS
4. EACH ROUND PLAYERS:
   - A. ROLL THE DICE TO DETERMINE SKILL
   - B. DECLARE SKILL SCORE ON TOP CARD
   - C. HIGHEST SCORE TAKES THE OTHER CARDS
   - D. IN CASE OF TIE: DECLARE JOKER SKILL SCORE
5. END WHEN ONE PLAYER HAS NO CARD LEFT

Creativity
Quantitative skills
Field & lab techniques
Administration skills
Communication skills
Teaching & mentoring
Interpersonal skills

Creativity
Quantitative skills
Field & lab techniques
Administration skills
Communication skills
Teaching & mentoring
Interpersonal skills

Creativity
Quantitative skills
Field & lab techniques
Administration skills
Communication skills
Teaching & mentoring
Interpersonal skills

Creativity
Quantitative skills
Field & lab techniques
Administration skills
Communication skills
Teaching & mentoring
Interpersonal skills

Creativity
Quantitative skills
Field & lab techniques
Administration skills
Communication skills
Teaching & mentoring
Interpersonal skills

FRONT SIDE

- Creativity
- Quantitative skills
- Field & lab techniques
- Administration skills
- Communication skills
- Teaching & mentoring
- Interpersonal skills

- Creativity
- Quantitative skills
- Field & lab techniques
- Administration skills
- Communication skills
- Teaching & mentoring
- Interpersonal skills

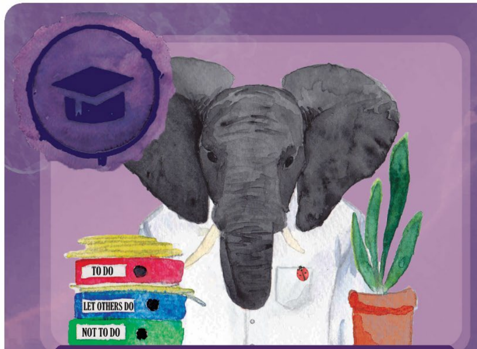

Coordinator

Head of administration,
Science officer, Research manager,
Coordinator of research project or study program

Required Skills

Creativity	★★★
Quantitative skills	★
Field & lab techniques	★
Administration skills	★★★★
Communication skills	★★★★
Teaching & mentoring	★★
Interpersonal skills	★★★★

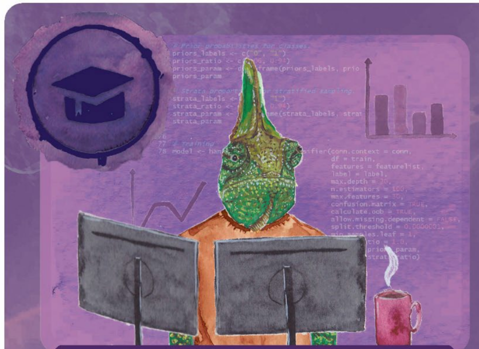

Data Scientist

Biostatistician, Bioinformatician,
Data analyst, Data manager, Data steward,
Data technician, Scientific programmer

Required Skills

Creativity	★★★
Quantitative skills	★★★★★
Field & lab techniques	★
Administration skills	★★
Communication skills	★★★★
Teaching & mentoring	★★
Interpersonal skills	★★★★

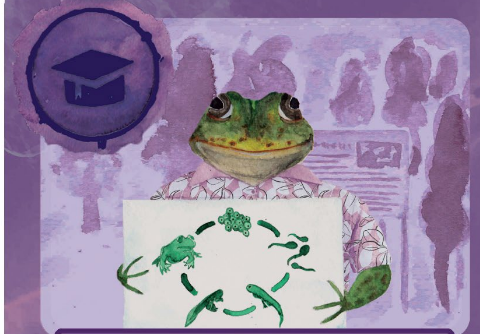

Educator

Lecturer at university or college

Required Skills

Creativity	★★★
Quantitative skills	★★★
Field & lab techniques	★★★
Administration skills	★★
Communication skills	★★★★★
Teaching & mentoring	★★★★★
Interpersonal skills	★★★★★

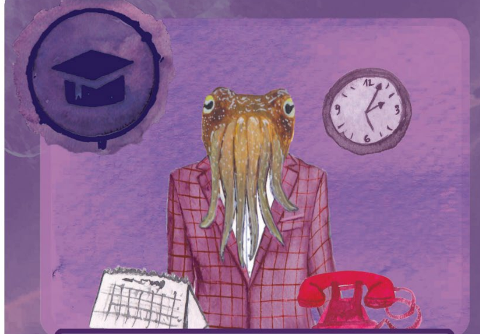

Manager

Field station manager, Project manager,
Laboratory technical manager,
Collection manager, Financial manager

Required Skills

Creativity	★★
Quantitative skills	★
Field & lab techniques	★★
Administration skills	★★★
Communication skills	★★★
Teaching & mentoring	★
Interpersonal skills	★★★

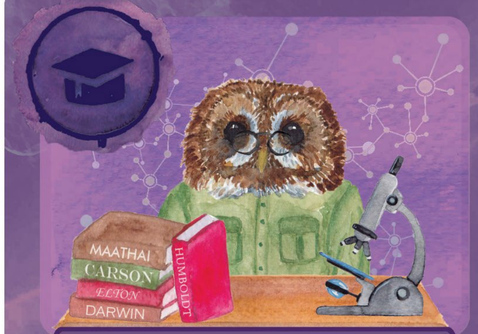

Researcher

Professor, Scientist, Postdoc at university,
college or public research institute

Required Skills

Creativity	★★★★
Quantitative skills	★★★★
Field & lab techniques	★★★
Administration skills	★★
Communication skills	★★★★
Teaching & mentoring	★★★
Interpersonal skills	★★★

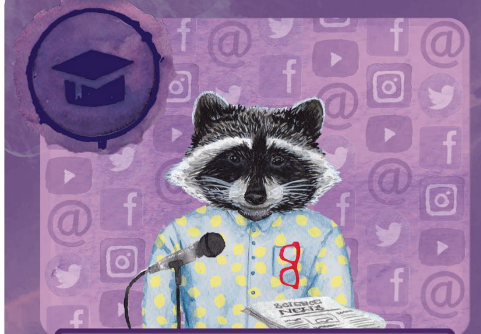

Science Communicator

Science communicator,
Public relations officer,
Content developer

Required Skills

Creativity	★★★★★
Quantitative skills	★★
Field & lab techniques	★
Administration skills	★★★
Communication skills	★★★★★
Teaching & mentoring	★★
Interpersonal skills	★★★★

FRONT SIDE

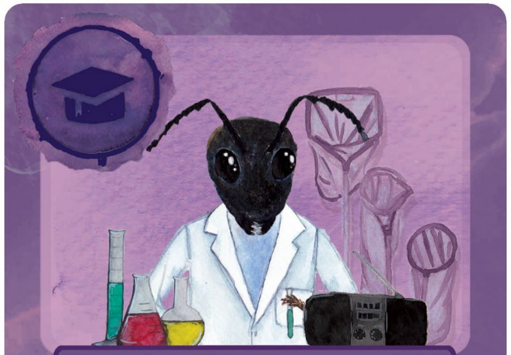

Technician

Lab technician,
Field technician,
Research engineer

Required Skills

Creativity	★★★
Quantitative skills	★
Field & lab techniques	★★★★★
Administration skills	★★★
Communication skills	★★★
Teaching & mentoring	★★
Interpersonal skills	★★★

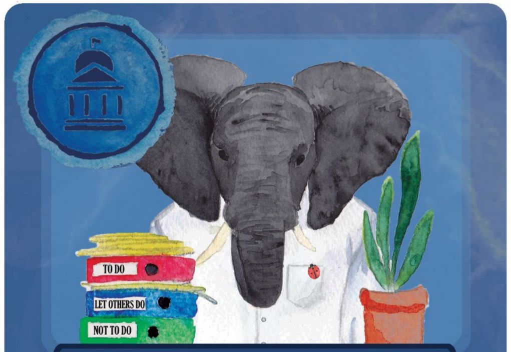

Coordinator

Project coordinator at governmental organization,
Funding coordinator/administrator

Required Skills

Creativity	★★★★
Quantitative skills	★★
Field & lab techniques	★
Administration skills	★★★
Communication skills	★★★★
Teaching & mentoring	★★
Interpersonal skills	★★★★

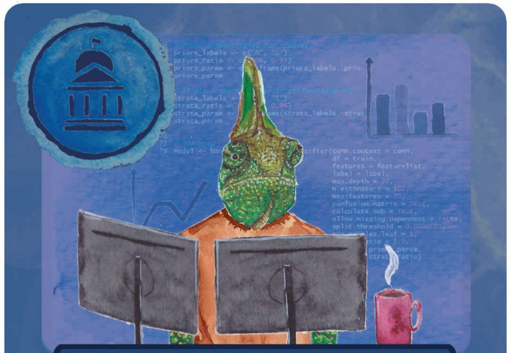

Data Scientist

Data analyst, Data steward,
Data scientist,
GIS analyst

Required Skills

Creativity	★★★★
Quantitative skills	★★★★★
Field & lab techniques	★
Administration skills	★
Communication skills	★★★
Teaching & mentoring	★
Interpersonal skills	★★★

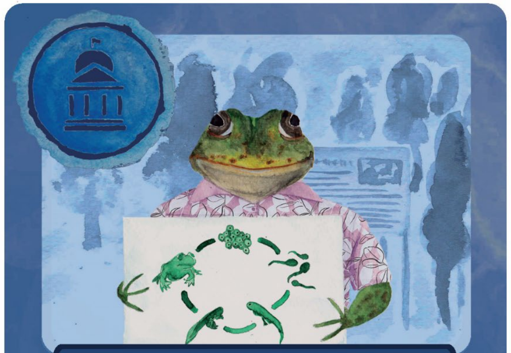

Educator

Teacher at a public school,
Educator in zoo, aquarium
or natural history museum

Required Skills

Creativity	★★★★★
Quantitative skills	★
Field & lab techniques	★
Administration skills	★★★
Communication skills	★★★★★
Teaching & mentoring	★★★★★
Interpersonal skills	★★★★

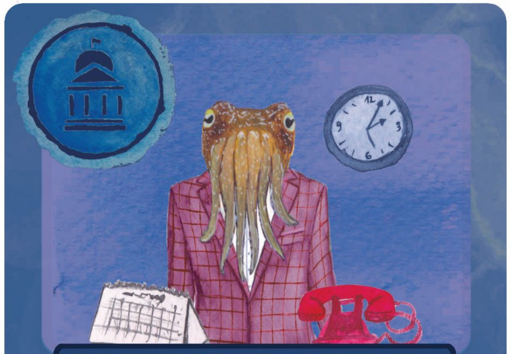

Manager

Conservation manager, Restoration manager,
Land resource manager, Collection manager,
Laboratory manager, Education manager

Required Skills

Creativity	★★★
Quantitative skills	★
Field & lab techniques	★★
Administration skills	★★★
Communication skills	★★★★
Teaching & mentoring	★★
Interpersonal skills	★★★

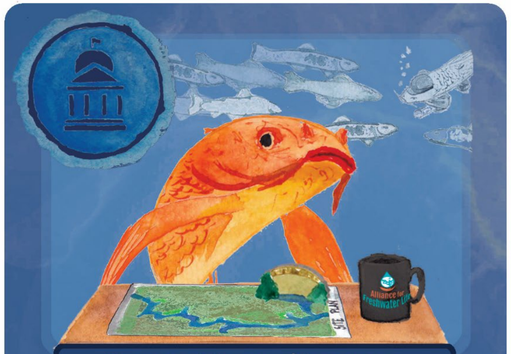

Policy Maker/Advisor

Ecological advisor, City ecologist,
Environmental consultant,
Science-policy officer

Required Skills

Creativity	★★
Quantitative skills	★★
Field & lab techniques	★★★
Administration skills	★★
Communication skills	★★★★★
Teaching & mentoring	★
Interpersonal skills	★★★

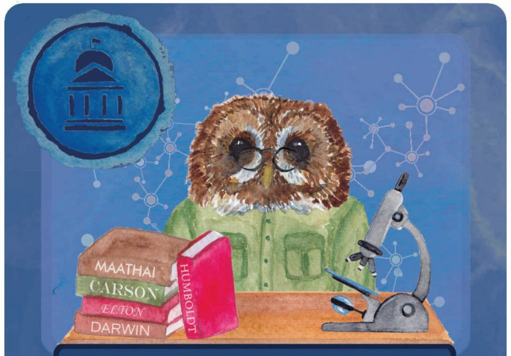

Researcher

Researcher at governmental organization,
Museum curator

Required Skills

Creativity	★★★
Quantitative skills	★★★
Field & lab techniques	★★★★★
Administration skills	★★
Communication skills	★★★★
Teaching & mentoring	★★
Interpersonal skills	★★★

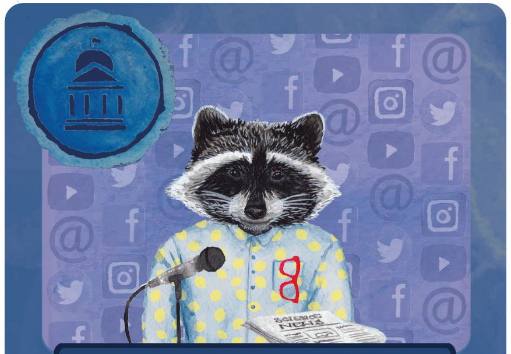

Science Communicator

Communication advisor,
Public information officer,
Public relations officer

Required Skills

Creativity	★★★★
Quantitative skills	★
Field & lab techniques	★
Administration skills	★★★
Communication skills	★★★★★
Teaching & mentoring	★★
Interpersonal skills	★★★★★

FRONT SIDE

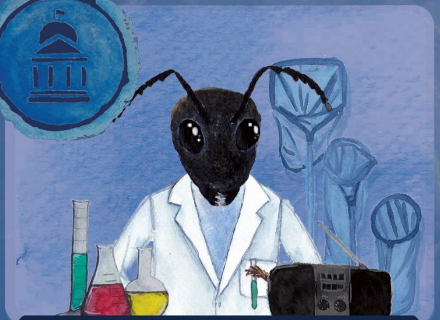

Technician

Field ecologist,
Field technician,
Lab technician

Required Skills

Creativity	★★★
Quantitative skills	★
Field & lab techniques	★★★★★
Administration skills	★
Communication skills	★★★
Teaching & mentoring	★
Interpersonal skills	★★★★

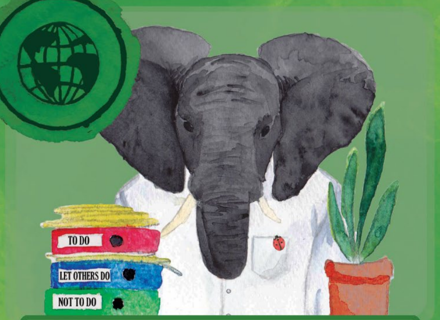

Coordinator

Project coordinator at NGO (e.g. environmental education, conservation, monitoring, experiments), Funding coordinator

Required Skills

Creativity	★★
Quantitative skills	★★
Field & lab techniques	★
Administration skills	★★★★
Communication skills	★★★★★
Teaching & mentoring	★★
Interpersonal skills	★★★★★

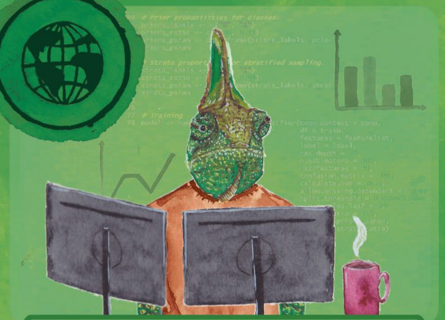

Data Scientist

General data scientist,
Environmental informatician,
GIS analyst

Required Skills

Creativity	★★
Quantitative skills	★★★★★
Field & lab techniques	★★
Administration skills	★★
Communication skills	★★★
Teaching & mentoring	★
Interpersonal skills	★★

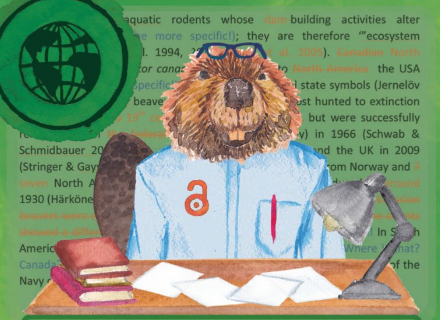

Editor/Publisher

Editor/publisher at NGO
including scientific societies
(e.g. books, reports, journals, websites)

Required Skills

Creativity	★★★
Quantitative skills	★
Field & lab techniques	★
Administration skills	★★★★
Communication skills	★★★★
Teaching & mentoring	★★★
Interpersonal skills	★★★★

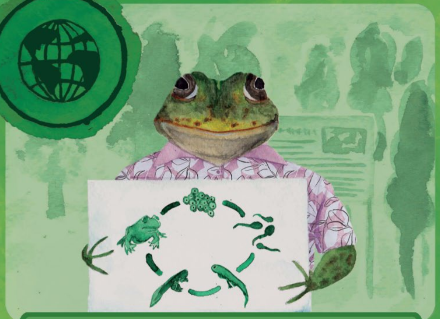

Educator

Environmental educator (e.g. in collaboration with educational institutions like schools/universities), Guide in conservation areas

Required Skills

Creativity	★★★★
Quantitative skills	★
Field & lab techniques	★
Administration skills	★★
Communication skills	★★★★★
Teaching & mentoring	★★★★
Interpersonal skills	★★★★

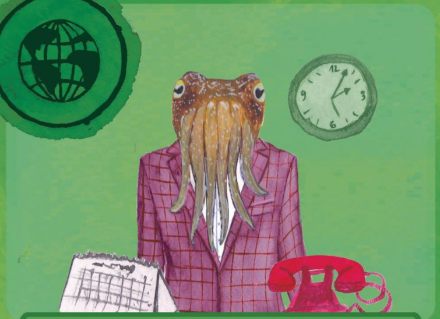

Manager

Project manager at NGO (e.g. environmental education, conservation, monitoring), Nature park manager, Conservation officer

Required Skills

Creativity	★★★
Quantitative skills	★★
Field & lab techniques	★★
Administration skills	★★
Communication skills	★★★★
Teaching & mentoring	★★★
Interpersonal skills	★★★★

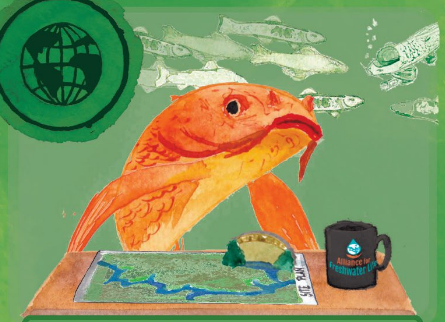

Policy Maker/Advisor

Policy advisor (science diplomacy, regional or international policy, environmental law), Lobbyist

Required Skills

Creativity	★★
Quantitative skills	★★★★
Field & lab techniques	★★★★
Administration skills	★★
Communication skills	★★★★
Teaching & mentoring	★
Interpersonal skills	★★★

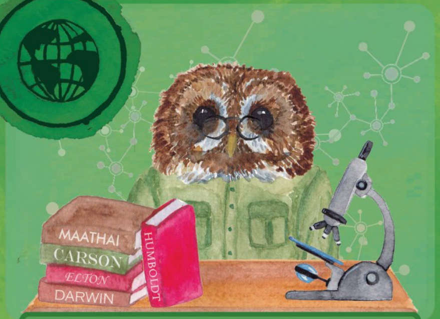

Researcher

Researcher at non-governmental organization (NGO)

Required Skills

Creativity	★★★★
Quantitative skills	★★★★★
Field & lab techniques	★★★★
Administration skills	★★★
Communication skills	★★★★
Teaching & mentoring	★★
Interpersonal skills	★★★★

FRONT SIDE

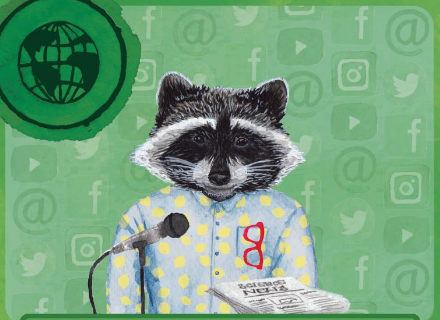

Science Communicator

Social media/Public relations or Marketing for non-governmental organization (NGO) including scientific societies

Required Skills

Creativity	★★★★
Quantitative skills	★★★
Field & lab techniques	★★★
Administration skills	★★★
Communication skills	★★★★
Teaching & mentoring	★★★
Interpersonal skills	★★★★★

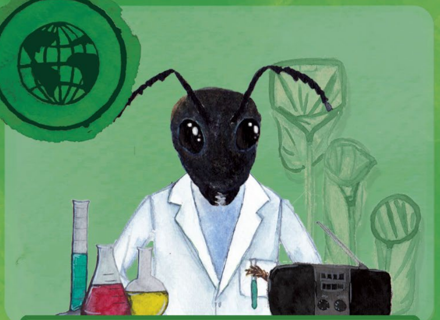

Technician

Laboratory or field technician at non-governmental organization (NGO)

Required Skills

Creativity	★★★
Quantitative skills	★★
Field & lab techniques	★★★★★
Administration skills	★
Communication skills	★★★
Teaching & mentoring	★
Interpersonal skills	★★★

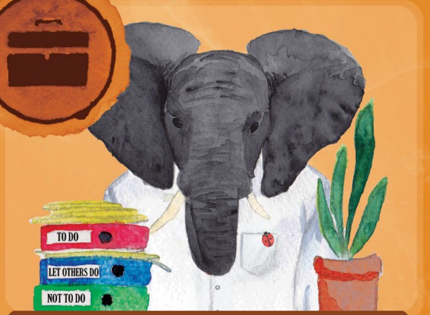

Coordinator

Project coordinator at private company/start-up, Entrepreneur, Coordinator for funding, ecological monitoring or conservation volunteers

Required Skills

Creativity	★★★
Quantitative skills	★
Field & lab techniques	★★★
Administration skills	★★★
Communication skills	★★★★
Teaching & mentoring	★
Interpersonal skills	★★★

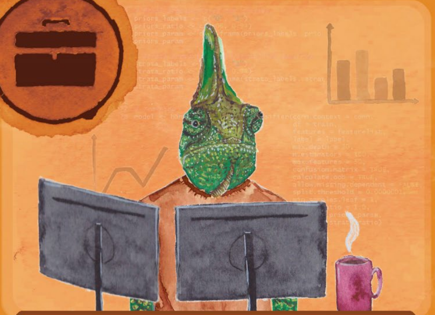

Data Scientist

Environmental informatician, GIS analyst, Research and development analyst, Data analyst, Software developer

Required Skills

Creativity	★★★
Quantitative skills	★★★★★
Field & lab techniques	★
Administration skills	★
Communication skills	★★★
Teaching & mentoring	★★
Interpersonal skills	★★

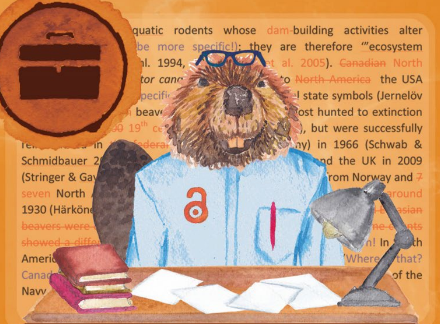

Editor/Publisher

Editor or Copy editor (e.g. scientific books, private scientific journals or popular science magazines)

Required Skills

Creativity	★★
Quantitative skills	★
Field & lab techniques	★
Administration skills	★★★★
Communication skills	★★★★
Teaching & mentoring	★
Interpersonal skills	★★★

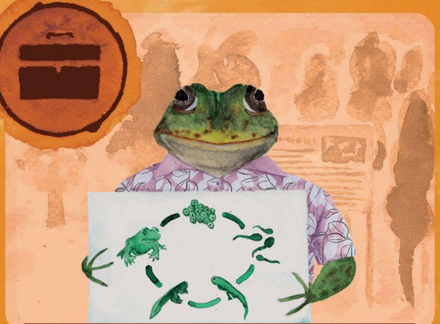

Educator

Educator in zoo, aquarium or natural history museum, Biology teacher (private schools, freelance), Environmental educator, Nature guide

Required Skills

Creativity	★★★
Quantitative skills	★
Field & lab techniques	★
Administration skills	★★
Communication skills	★★★★★
Teaching & mentoring	★★★★★
Interpersonal skills	★★★★★

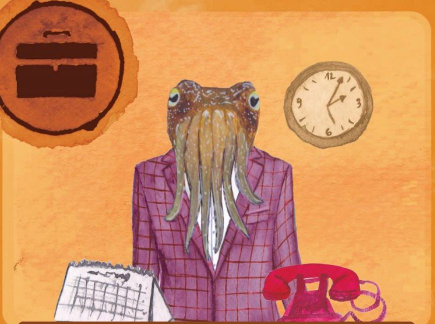

Manager

Restoration manager, Natural resource manager, Quality assurance manager, Operations manager, Zoo or aquarium manager

Required Skills

Creativity	★★★★
Quantitative skills	★
Field & lab techniques	★
Administration skills	★★★★
Communication skills	★★★★★
Teaching & mentoring	★
Interpersonal skills	★★★★

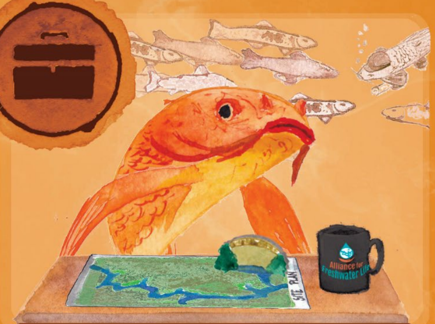

Policy Maker/Advisor

Environmental or sustainability consultant, Business advisor

Required Skills

Creativity	★★
Quantitative skills	★★★
Field & lab techniques	★★★★
Administration skills	★★★
Communication skills	★★★
Teaching & mentoring	★
Interpersonal skills	★★★★

FRONT SIDE

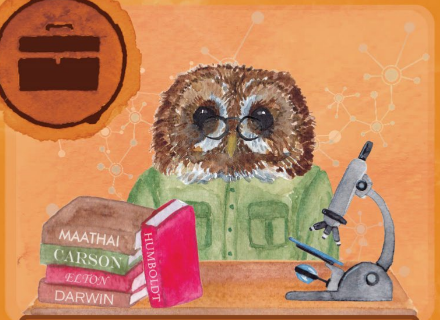

Researcher

Researcher at private company (e.g. biotechnology, agriculture, health & epidemiology, environment, hydrobiology), Museum curator

Required Skills

Creativity	★★★★
Quantitative skills	★★★★
Field & lab techniques	★★★★★
Administration skills	★★
Communication skills	★★★★
Teaching & mentoring	★★★
Interpersonal skills	★★★★

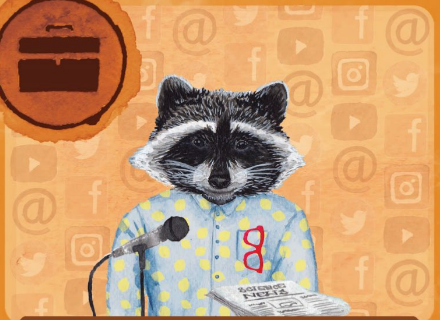

Science Communicator

Content developer, Medical/technical writer, Popular science writer, Science journalism, Marketing for ecotourism

Required Skills

Creativity	★★★★
Quantitative skills	★
Field & lab techniques	★
Administration skills	★★★
Communication skills	★★★★★
Teaching & mentoring	★
Interpersonal skills	★★★★★

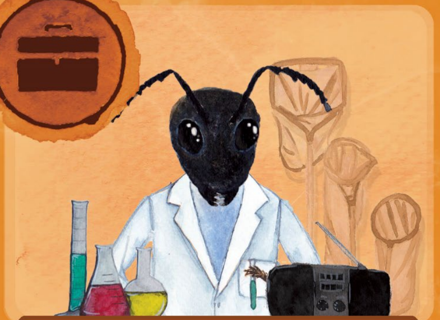

Technician

Field technician (e.g. ecological surveys), Laboratory technician, Medical technician, Animal care technician, Veterinary technician

Required Skills

Creativity	★★
Quantitative skills	★
Field & lab techniques	★★★★★
Administration skills	★★
Communication skills	★★★
Teaching & mentoring	★
Interpersonal skills	★★

BACK SIDE

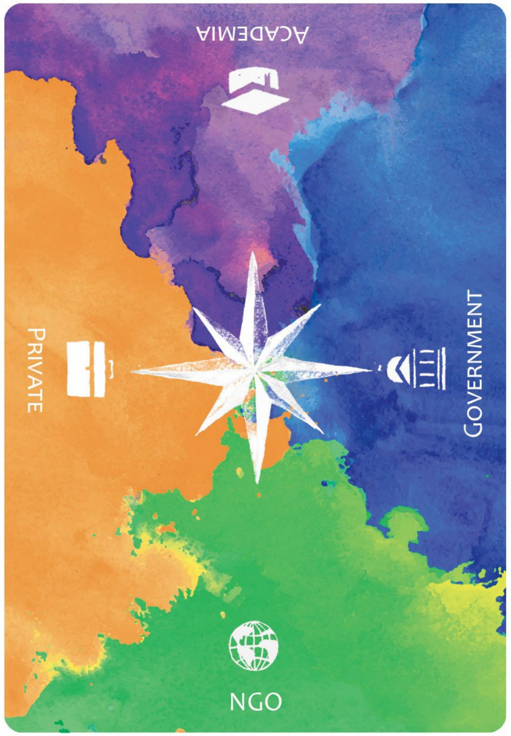
ACADEMIA
PRIVATE
GOVERNMENT
NGO

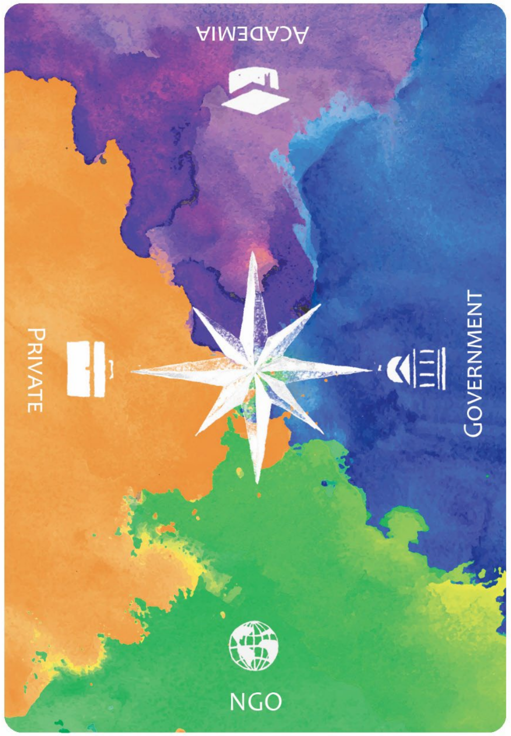
ACADEMIA
PRIVATE
GOVERNMENT
NGO

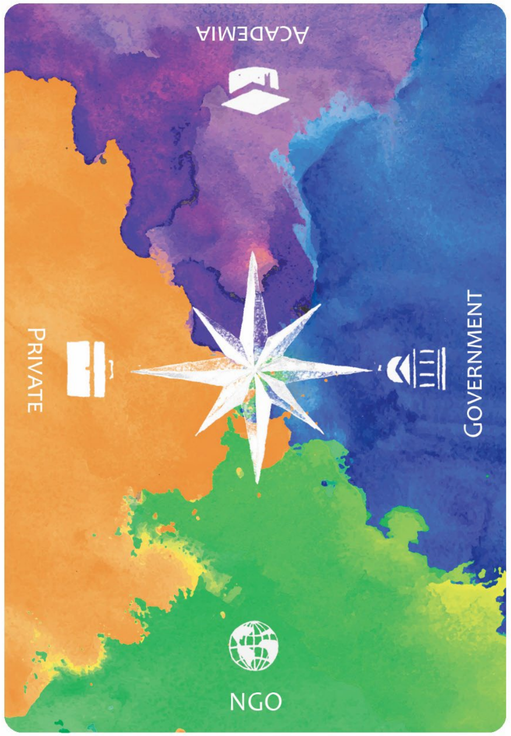
ACADEMIA
PRIVATE
GOVERNMENT
NGO

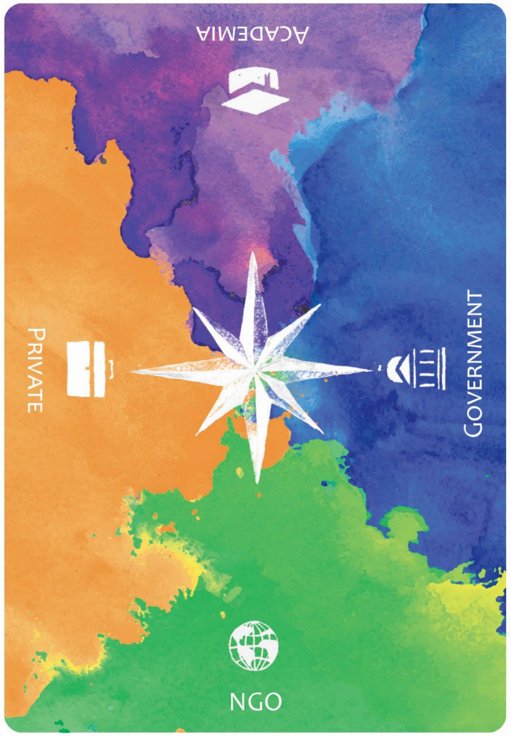
ACADEMIA
PRIVATE
GOVERNMENT
NGO

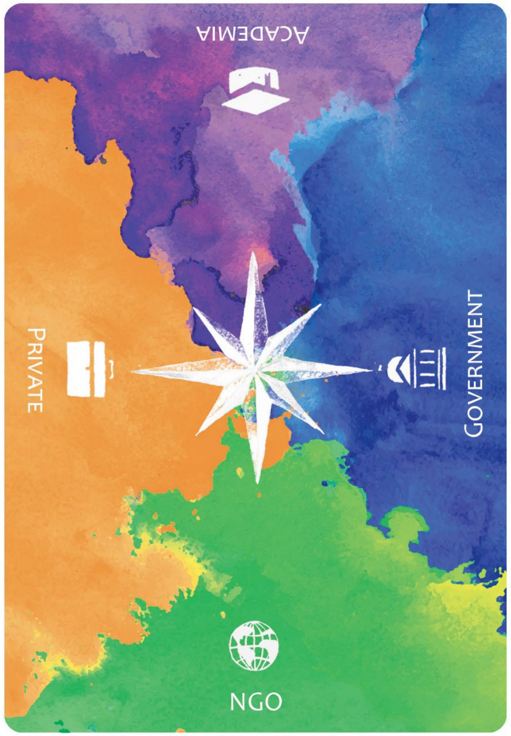
ACADEMIA
PRIVATE
GOVERNMENT
NGO

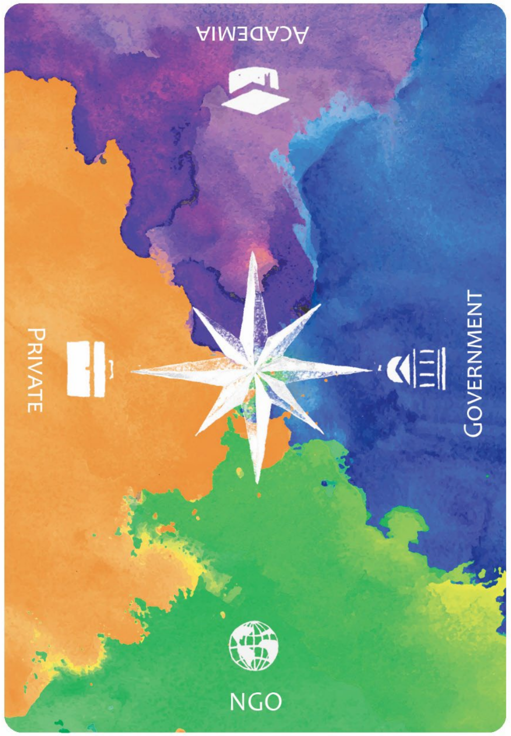
ACADEMIA
PRIVATE
GOVERNMENT
NGO

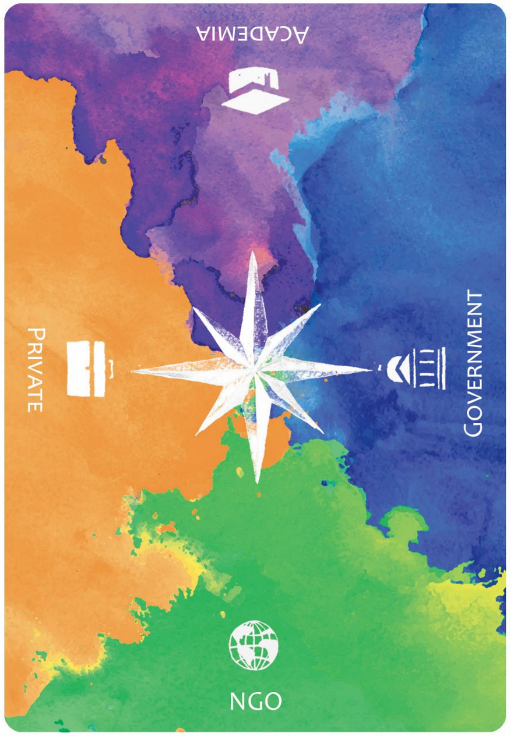
ACADEMIA
PRIVATE
GOVERNMENT
NGO

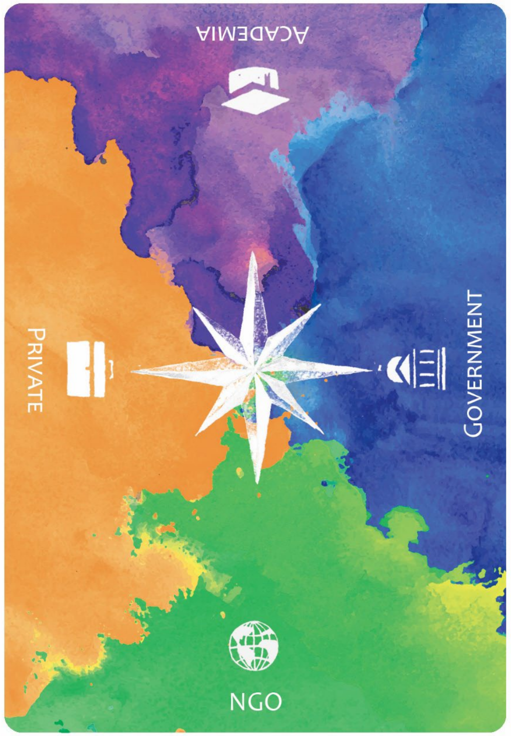
ACADEMIA
PRIVATE
GOVERNMENT
NGO
